# Development and Potent Anti-Tumor Activity of a Fully Humanized Anti-TAG-72-IL-2 Fusion Protein for Therapy of Solid Tumors

**DOI:** 10.3390/cancers17091453

**Published:** 2025-04-26

**Authors:** Eric Aniogo, Lindsay Williams, Teresa Hong, Patty Wong, Susanta K. Hui, Hemendra Ghimire, Erasmus K. Poku, David M. Colcher, Paul J. Yazaki, John E. Shively, Maciej Kujawski

**Affiliations:** 1 Department of Immunology and Theranostics, Beckman Research Institute, City of Hope, Duarte, CA 91010, USA; eaniogo@coh.org (E.A.); lindsaywilliams260@gmail.com (L.W.); tehong@coh.org (T.H.); pmwong@coh.org (P.W.); dcolcher@coh.org (D.M.C.); pyazaki@coh.org (P.J.Y.); jshively@coh.org (J.E.S.); 2 Department of Radiation Oncology, Beckman Research Institute and Medical Center, City of Hope, Duarte, CA 91010, USA; shui@coh.org (S.K.H.); heghimire@coh.org (H.G.); 3 Radiopharmacy, Beckman Research Institute, City of Hope, Duarte, CA 91010, USA; kpoku@coh.org

**Keywords:** TAG-72, fusion proteins, colon cancer, breast cancer, radiation therapy, immunotherapy

## Abstract

**Background/Objectives:** Immunocytokines (ICKs) are antibody–cytokine fusion proteins that deliver cytokines directly to tumors to increase immune responses, which are otherwise absent or limited by the delivery of antibodies alone. Tumor-associated glycoprotein-72 (TAG72) is overexpressed in numerous solid tumors. **Methods:** An anti-TAG72-IL-2 fusion protein was expressed in mammalian cells and tested in vitro for binding and bioactivity, and in vivo in two models. **Results:** In vitro studies showed high antigen specificity against TAG-72-positive tumor cell lines and IL-2 activity in CD25 (IL-2R)-positive reporter cells. To study the anti-tumor activity of huCC49-IL-2 in an immunocompetent model, the TAG-72 expression was established in murine mammary and colorectal cells by transfection with murine st6galnac-I gene (mSTn). Four daily doses of anti-TAG72-IL-2 monotherapy for TAG-72-expressing orthotopic murine mammary tumors in immunocompetent mice resulted in minimal whole-body toxicity and significant tumor growth reduction mediated by tumor infiltration of IFNγ^+^ CD8^+^ T cells. When mammary tumors were pretreated with image-guided fractionated radiation therapy (IGRT) followed by anti-TAG72-IL-2 therapy, an improved tumor growth inhibition was observed along with an increased tumor infiltration of IFNγ^+^ CD8^+^ T cells and a significant reduction in Foxp3^+^ CD4^+^ cells. Anti-TAG72-IL-2 monotherapy in TAG-72^+^ colorectal tumors led to a significant tumor reduction but also cures (4/7), with a rejection of rechallenges with both TAG-72-positive and -negative MC38 cells, thus demonstrating evidence of immune memory and antigen spreading. **Conclusions:** antiTAG-72-IL-2 therapy showed strong anti-tumor effects driven by activated CD8^+^ T cells making it a promising approach to the treatment of TAG-72^+^ tumors.

## Simple Summary

Tumor-associated glycoprotein-72 (TAG-72), an aberrant glycosylated cell surface glycoprotein molecule, is overexpressed in several epithelial cancers. The antibody–cytokine fusion proteins called immunocytokines (ICKs) were designed to boost anti-tumor immunity. In the current study, we demonstrated the engineering, production, and bioactivity of a fully humanized anti-TAG-72-IL-2 fusion protein (huCC49-IL-2). The in vivo testing in animal models showed potent anti-tumor immune responses driven by the recruitment of activated and cytotoxic CD8^+^ T cells to the tumors. The colorectal carcinoma model treatment with huCC49-IL-2 eliminated a majority of the primary tumors and led to the establishment of immune memory and antigen spreading. Our new molecule could represent a promising new tool in the treatment of solid tumors.

## 1. Background

Many tumor-specific antibodies are poorly cytotoxic or fail to generate an immune response in tumors due to their inherent immunosuppressive microenvironment. One approach to boosting their activity is to engineer antibody–cytokine fusion proteins, called immunocytokines (ICKs). To prevent off-target tissue toxicity, it is important to select a target antigen with limited expression in normal cells. Among the possible candidates, tumor-associated glycoprotein-72 (TAG-72), an aberrant glycosylated cell surface glycoprotein molecule overexpressed in colon, breast, ovary, and stomach cancers, stands out for its limited normal tissue expression [1,2,3]. For example, TAG-72 shows low expression in secretory endometrial tissue and duodenal goblet cells, making it an attractive target for cancer immunotherapy, including CAR-T therapy [4,5]. TAG-72 has important immunosuppressive effects on tumor-infiltrating dendritic cells [6]. Hence, TAG-72-specific antibodies have been generated for cancer detection as well as for targeting molecules for tumor therapy. Among these antibodies, CC49 shows a high specificity and affinity for TAG-72 [7,8]. Both murine and chimeric formats of CC49 were used in positron emission tomography (PET) for the in vivo detection of a wide range of solid tumors [9,10]. Although CC49 has limited efficacy as a single antibody, a CC49 antibody–drug conjugate (ADC) carrying a cytotoxic payload delayed tumor development and enhanced survival in an animal model [11]. CC49 radiolabeled with therapeutic radionuclides, such as Yttrium-90 or Iodine-131, has also exhibited significant anti-tumor effects in a variety of solid tumors [12,13]. For example, we recently showed that ^225^Ac-labeled huCC49 in tumor-targeted alpha therapy (TAT) significantly reduced tumor growth in a dose-dependent manner with low off-target toxicity [14].

The idea of altering the immunosuppressive environment of solid tumors by the antibody-mediated delivery of immunomodulatory ICKs has shown promise as another approach. These genetically engineered antibodies are generated by linking tumor-reactive monoclonal antibodies to cytokines that augment the immune response [15,16,17].

Interleukin-2 (IL-2, Proleukin^®^) is one of the first immunotherapies used to treat metastatic renal cell carcinoma and melanoma. IL-2 is a potent stimulator of the immune system that activates and maintains T-effector (T_eff_) and natural killer (NK) cells. However, systemic high-dose IL-2 often produces dose-limiting toxicities (DLTs), the most severe being vascular leak syndrome (VLS) and the stimulation of immunosuppressive CD4^+^ FoxP3^+^ T-regulatory (T_reg_) cells. To expand the clinical utility of IL-2, there has been a successful advance in separating the IL-2 receptor signaling of therapeutic effects from off-tumor effects [18,19,20,21].

To address these problems, ICKs with interleukin-2 (IL-2) as the antibody fusion partner have been developed and shown to retain good tumor targeting while stimulating a localized anti-tumor response [22,23]. For example, human-IL-2 linked to tumor-reactive mAbs such as hu14.18-IL-2, anti-CD20-IL-2, DI-Leu16-IL-2, and anti-CEA M5A-IL-2 have shown similar trends of stimulating an antibody-directed response against cancer, including testing for cancer therapy currently in clinical trials [15,24].

In the current study, we demonstrate the engineering, production, and bioactivity of a fully humanized anti-TAG-72-IL-2 fusion protein. The developed huCC49-IL-2 exhibited high antigen specificity and activity in vitro. The targeting of lymphoid organs and TAG-72-positive tumors was confirmed in vivo by PET imaging. Moreover, in two syngeneic animal models, we showed potent anti-tumor immune responses driven by the recruitment of cytotoxic CD8^+^ T cells to the tumor. Furthermore, in an MC38 murine model of colorectal cancer, the potent elimination of primary tumors was observed in a monotherapy approach, including the eradication of tumor rechallenge with both TAG-72-positive and -negative MC38 cells, thus demonstrating evidence of immune memory and antigen spreading.

## 2. Materials and Methods

### 2.1. Generation, Expression, and Purification of huCC49-IL-2 Fusion Protein

The genetic maps of the heavy and light chains of huCC49 (pEE12.4 huCC49 L chain and pEE6.4-huCC49 H chain-huIL-2, respectively), as described by Larson et al. [25], and protein sequences are shown in Supplementary Figure S1. The huCC49 light chain (kappa) sequence was inserted into the pEE12.4 plasmid vector flanked by the HindIII cut site, Kozak sequence, VL signal sequence on the 5′ end and 2× stop codons, and EcoRI cut site at the 3′ end. The canonical human IL-2 sequence (on 3′ of the H chain) was inserted into the pEE6.4 plasmid vector, followed by 2× stop codons and the EcoRI cut site, with the 5′ end containing the Kozak sequence and VH signal sequence, followed by the huCC49 heavy chain. The synthetic genes were purchased from GeneArt (ThermoFisher, Waltham, MA, USA) and propagated in NEB5α™ cells (New England Biolabs, Ipswich, MA, USA), sub-cloned into the expression vectors pEE6.4 and pEE12.4 (Lonza Biologics, Basel, Switzerland), and verified by Sanger sequencing.

Transient transfection was performed using the Expi293F™ mammalian expression system (ThermoFisher, Waltham, MA, USA). Two milliliters of cells were transfected with 2 µg of plasmid at the following ratios of HC to LC: 1:1, 1:2, 1:3, 2:1, and 3:1, or with no DNA as a negative control. On day 6, the supernatants were harvested, 0.22 µm PES filtered and clarified by batch treatment (5% *w*/*v*) with the anion exchanger AG1×8 (Bio-Rad Laboratories, Hercules, CA, USA), followed by running 15 µL aliquots on a denaturing non-reducing NuPAGE 4–12% Bis-Tris SDS-PAGE gel (ThermoFisher) (run in 1× MOPS SDS Running Buffer) to determine the highest expression ratio.

Further scaled-up transfections were performed at this ratio, and the product was captured on a 5 mL HiTrap Protein A column (Cytiva™, Marlborough, MA, USA) using PBS for the column equilibration and loading, followed by 5–7 column volumes of high salt buffer wash (20 mM sodium phosphate dibasic, 20 mM sodium citrate, 500 mM NaCl, pH 7.5) and 2–5 column volumes of PBS, and then eluted with 0.5 M Arginine (pH 4.02). Arginine was used to stabilize the protein after the elution to prevent precipitation at a low pH. The samples were neutralized using 1 M Tris-Cl (pH 8.0, titrated to pH 6.0) and dialyzed on a 10 kDa Slide-a-Lyzer™ dialysis cassette (ThermoFisher) in PBS (5 × 5 L). The dialyzed samples were concentrated using 10 K Amicron^®^ Ultra centrifugal filters (Millipore Sigma, Burlington, MA, USA) in PBS, sterile filtered, and stored at 4 °C. The huCC49-IL-2 concentrated products were further purified using a ceramic hydroxyapatite (CHT) type I column (BioRad) to remove protein aggregates and then subjected to HPLC size exclusion chromatography (SEC) using a Superdex 200 column (Cytiva) to check the purity with the elution at ~11.5 mL, as expected for a protein of this size. After the purification, dialysis, and concentration, the scaled-up yield was ~200 µg/mL based on the transfection of 270 µg of plasmid (1:3 HC:LC in 270 mL total of Expi293F cells).

### 2.2. Immunological Characterization and Activity Measurement of huCC49-IL-2

The immunoreactivity of huCC49-IL-2 was compared with that of M5A-IL-2 ICK on HEK-Blue™ IL-2 reporter cells and analyzed using a CLARIOstar plate reader (BMG Labtech, Cary, NC, USA). The IL-2 activity was measured using a HEK-Blue™ IL-2 reporter cell line (InvivoGen, San Diego, CA, USA), which expressed the inducible secreted embryonic alkaline phosphatase (SEAP) after the IL-2 receptor activation. The cells (5 × 10^4^ cells per well) were stimulated with huCC49-IL-2- or M5A-IL-2, and the supernatant was collected after 20 h and then developed with the QUANTI-Blue (InvivoGen) colorimetric enzyme assay reagent at 37 °C according to the manufacturer’s instructions. The absorbance was measured at 630 nm after 1 h using a CLARIOstar plate reader (BMG Labtech, Cary, NC, USA).

### 2.3. Cell Lines

Human ovarian carcinoma OV90, murine breast cancer E0771 (obtained from Dr. Saul Priceman, City of Hope, Duarte, CA, USA), and MC38 colon cancer (obtained from Dr. Steven A. Rosenberg, National Cancer Institute, Bethesda MD, USA) cell lines were grown in Dulbecco’s modified DMEM medium (DMEM; Gibco) containing 10% FBS, L-glutamine, and 100 U/mL penicillin/streptomycin. Both the E0771 and MC38 cells were stably transfected with the target antigen TAG-72 via transduction with an epHIV7 lentivirus carrying the murine st6galnac-I gene (mSTn) under the control of EF1α, as previously described [4]. The cell cultures were tested annually for the presence of mycoplasma using a mycoplasma detection kit (Universal Mycoplasma Detection Kit; ATCC).

### 2.4. Animal Studies

All animal experiments were performed using immunocompetent C57BL/6J mice (The Jackson Laboratory). For the breast carcinoma model, E0771/TAG72 tumor cells were injected into the mammary fat pad at a concentration of 1 × 10^5^ cells in PBS/Matrigel (1:1 ratio; total volume, 50 µL). The mice were randomized into control and treated groups, and the average tumor volume was 100 mm^3^ (the tumor sizes were measured using a digital caliper and the volumes were calculated using the formula T_volume_ = length × width × height). In some experiments, the tumors were treated with 4 × 2.5 Gy daily fractions of external beam radiation starting at day 8 post tumor injection, using the Precision X-RAD SMART Plus/225cx (Precision X-Ray, North Branford, CT, USA), as previously described [24]. Four daily doses of huCC49-IL-2 (1 mg/kg) were administered via intraperitoneal injections, starting from day 10 post tumor injection (for monotherapy) or day 12 (for combination with IGRT). The mice were humanely euthanized when the tumor volume exceeded 1500 mm^3^. For the colon cancer model, the MC38/TAG72 cells were injected subcutaneously at a concentration of 1 × 10^6^ cells (total volume, 50 µL). During the experiment, some of the mice received a second subcutaneous injection of MC38/TAG72 or MC38 parental cells at the same concentration on the opposite flank. The mice were randomized as described above. Four daily doses of huCC49-IL-2 (1 mg/kg) were administered via intraperitoneal injections, starting from day 11 post tumor injection. The experimental procedures were performed by EA, HG, and MK, and they were aware of the group allocations. The mouse-care and experimental procedures were performed under pathogen-free conditions in accordance with established institutional guidelines and protocols approved by the Institutional Animal Care and Use Committee of the Beckman Research Institute at the City of Hope National Medical Center (IACUC protocol 91037 approved on 12 September 2023).

### 2.5. PET Imaging Studies

Humanized anti-TAG-72 antibody huCC49 or huCC49-IL2 was conjugated with NHS-DOTA, as previously described [26]. The antibodies were radiolabeled with ^64^Cu at a ratio of 0.37:1 MBq/mg. The immunoreactivity of both antibodies against TAG-72 was confirmed, and animal PET imaging studies were performed in C57BL/6J mice bearing E0771/TAG72 tumors, as previously described [14]. The PET scans were conducted using MOLECUBES micro-PET and CT scanners (Molecubes Inc., Lexington, MA, USA). At the terminal time point, the mice were euthanized, and biodistribution studies were performed.

### 2.6. Leukocyte Analysis

Spleens, tumor-draining lymph nodes (TDLNs), and tumors were collected, prepared, and analyzed by flow cytometry, as described previously, using similar gating strategies [24]. Single cell suspensions were stained with different combinations of fluorochrome-coupled antibodies against CD3, CD4, CD8, B220, CD19, CD11b, Ly6C, Ly6G, CD11c, F4/80, NK1.1, NKp46, PD-1, CTLA-4, and Tim-3 (BioLegend, San Diego, CA, USA). The expression of IFNγ and FoxP3 was studied using intracellular flow cytometry, as described previously [24].

### 2.7. Statistical Analysis

To determine the statistical significance of the differences between the treatment groups, we used an unpaired *t*-test and a two-way ANOVA test. The *p*-values are indicated in the figures as follows: **** *p* < 0.0001, *** *p* < 0.001, ** *p* < 0.01, * *p* < 0.05. A survival analysis was performed using the log-rank (Mantel–Cox) test. The data were analyzed using the GraphPad Prism software (Version 10.3.1).

## 3. Results

**Figure 1:**
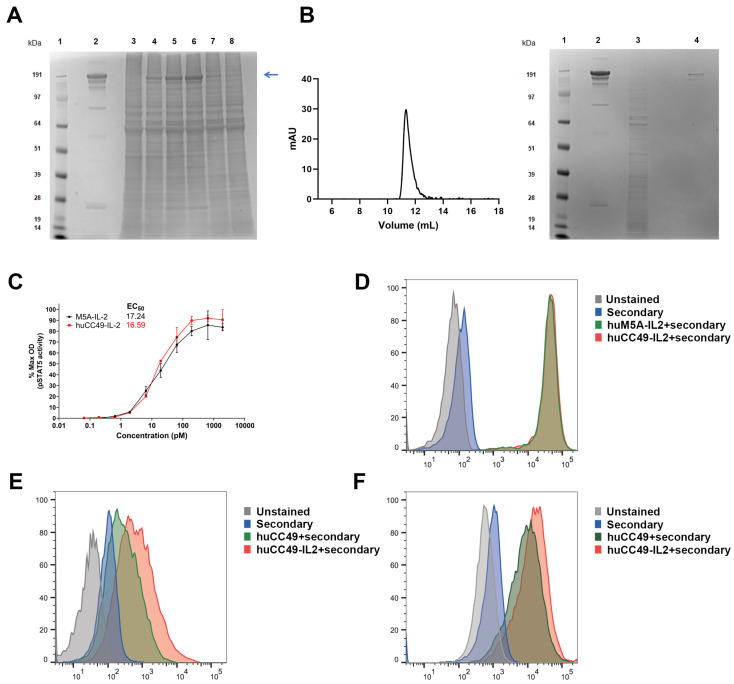
Generation and bioactivity of anti-TAG-72-IL-2 fusion protein. (**A**) Non-reducing SDS-PAGE stained with Coomassie Blue. Lanes: 1. MW markers; 2. M5A-IL2-ICK control; 3. 15 µL of harvest from negative (no DNA) culture; 4. 15 µL of harvest from culture transfected with heavy chain (HC) to light chain (LC) plasmid ratio 1:1; 5. HC:LC 1:2; 6. HC:LC 1:3; 7. HC:LC 2:1; 8. HC:LC 3:1. Band of interest (full length huCC49-IL-2 ICK) is indicated with blue arrow. (**B**) Left panel—size exclusion chromatogram of protein A and CHT purified huCC49-IL-2 (harvested from HC:LC ratio 1:3 scaled-up transfection from (**A**)). Right panel—non-reducing SDS-PAGE stained with Coomassie Blue. Lanes: 1. MW markers; 2. M5A-IL2-ICK control; 3. 15 µL of harvest from negative (no DNA) culture; 4. purified huCC49-IL-2. (**C**) huCC49-IL-2 IL-2 activity measured using HEK-Blue IL-2 reporter cell line. (**D**) Flow cytometry analysis of huCC49-IL-2 binding to HEK-Blue IL-2 reporter cells. (**E**) Flow cytometry analysis of huCC49-IL-2 binding to human ovarian carcinoma cell line OV90. (**F**) Flow cytometry analysis of huCC49-IL-2 binding to murine breast carcinoma E0771 transduced with murine st6galnac-I gene (mSTn) to express TAG-72. The original gels can be found in the Supplementary File S1.

The generation and bioactivity of an anti-TAG-72-IL-2 fusion protein, huCC49-IL-2, was engineered by the fusion of the gene for human IL-2 to the 3’ end of the gene of the heavy chain of huCC49 mAb. huCC49-IL-2, encoded in two Lonza plasmid vectors (pEE6.4 and pEE12.4) (Supplementary Figure S1A,B), was transfected according to the manufacturer’s protocol into Expi293F suspension cells. A splice overlap extension was used to mutate four of the last seven amino acid residues of the huCC49 heavy chain to eliminate a potential T-cell epitope and proteolytic cleavage site at the heavy chain/IL-2 junction. Several different ratios of heavy to light chain plasmid co-transfection were tested (1:1, 1:2, 1:3, 2:1, and 3:1), with the best results obtained using a 1:3 ratio (Figure 1A). The huCC49-IL-2 supernatant harvests were purified via protein A and CHT columns, followed by size exclusion (Superdex 200) and non-reducing SDS-PAGE to check for purity (Figure 1B, panels left and right, respectively). An elution at ~11.5 mL was expected for a protein of this molecular size. After the purification, dialysis, and concentration, a scaled-up 320 mL culture (~200 µg/mL) yielded a total of 55.8 mg. A high IL-2 activity of huCC49-IL-2 was observed in a bioassay (Figure 1C), with EC50 values similar to those of our anti-CEA M5A ICK [24].

Next, we tested the ability of huCC49-IL-2 to bind both IL-2 receptor-positive (HEK-Blue-CD25) and TAG-72-positive cells. Using flow cytometry, we found that huCC49-IL-2 showed high binding to the CD25-positive cells (Figure 1D), similar to our previously published anti-CEA M5A ICK [24]. The ability to bind the tumor antigen TAG-72 was tested using the human ovarian carcinoma cell line OV90. huCC49-IL-2 bound with an intensity similar to that of anti-TAG-72 huCC49 (Figure 1E). To study the anti-tumor activity of huCC49-IL-2 in an immunocompetent model, we generated murine carcinoma cell lines expressing the target antigen TAG-72 via transduction with an epHIV7 lentivirus carrying the murine st6galnac-I gene (mSTn) under the control of the EF1α promoter, as described by Lee et al. [4]. We confirmed the ability of huCC49-IL-2 to bind TAG-72 on the surface of murine breast carcinoma E0771/TAG72 cells with an intensity similar to that of the huCC49 antibody (Figure 1F).

**Figure 2:**
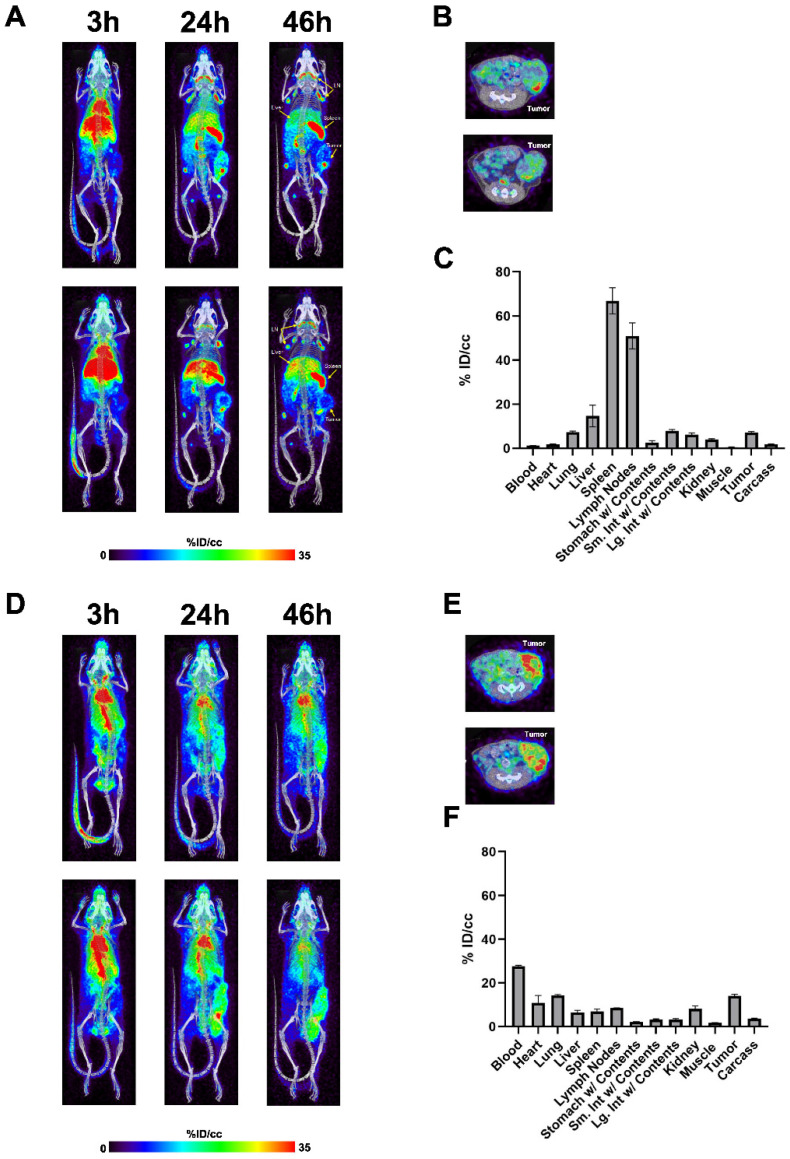
In vivo targeting of TAG-72-positive tumors. (**A**) PET imaging using ^64^Cu-labeled huCC49-IL-2. Imaging performed in breast carcinoma E0771/TAG72 tumor-bearing C57BL/6J female mice at 3, 24, and 46 h time points (coronal view) with lymph nodes, spleen, and tumor indicated by yellow arrows. (**B**) Transverse PET images of same mice at 46 h time point. (**C**) Biodistribution of ^64^Cu-labeled huCC49-IL-2 in indicated tissue shown as % of injected dose per gram of tissue at 46 h post injection (n = 3). (**D**) PET imaging using ^64^Cu-labeled intact huCC49. Imaging performed in breast carcinoma E0771/TAG72 tumor-bearing C57BL/6J female mice at 3, 24, and 46 h time points. (**E**) Transverse PET images of same mice at 46 h time point. (**F**) Biodistribution of ^64^Cu-labeled huCC49 in indicated tissue shown as % of injected dose per gram of tissue at 46 h post injection (n = 2).

For the in vivo tracking, targeting and biodistribution of the anti-TAG-72-IL-2 fusion protein to TAG-72-positive tumors, we conjugated huCC49-IL-2 with NHS-DOTA, radiolabeled with ^64^Cu, and performed immunoPET imaging. The PET imaging performed in the E0771/TAG72 tumor-bearing female mice showed fast blood clearance, with a predominant lymphoid tissue accumulation observed 24 h post injection (Figure 2A). At 46 h, we found tumor tissue targeting at 7% of the injected dose per gram (ID/g) by quantifying the PET images that were confirmed by the terminal biodistribution assessed by direct tissue counting (Figure 2B,C). The observed biodistribution was comparable to the values found in our previous anti-CEA IL-2 study [24]. Fast blood clearance and uptake in lymphoid organs such as the spleen and lymph nodes showed the most significant change in biodistribution compared with huCC49-IL-2 to intact huCC49 mAb. Compared to huCC49-IL-2, intact huCC49 mAb had high blood levels (25–30%ID/g %ID/g), with a tumor uptake of approximately 15% ID/g at 46 h (Figure 2D–F). The low blood levels demonstrated that huCC49-IL-2 altered the blood clearance compared to the intact antibody, a finding that needs further investigation to determine whether this would affect huCC49-IL-2’s anti-tumor immunity.

**Figure 3:**
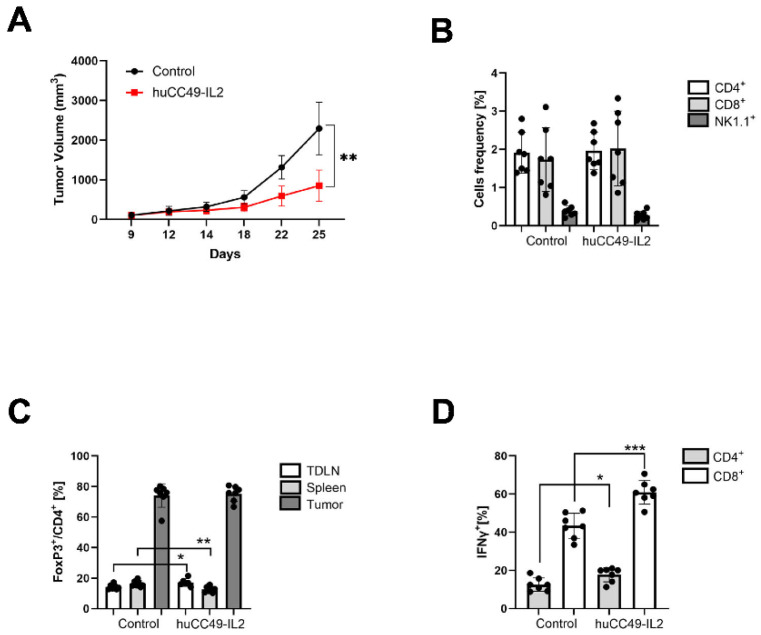
Potent anti-tumor activity against syngeneic murine breast carcinoma. (**A**) Inhibition of E0771/TAG72 tumor growth. Tumor-bearing C57BL/6J female mice were treated with four daily doses of huCC49-IL-2 (1 mg/kg) starting from day 10, when tumors reached 100 mm^3^ (n = 7 per group). (**B**) Flow cytometry analysis of tumor tissue for indicated populations shown as percentage of live cells (n = 7 per group). (**C**) Frequency of FoxP3^+^CD4^+^ T-regs in indicated tissues (n = 7 per group). (**D**) Frequency of IFNγ-expressing CD4^+^ and CD8^+^ T cells in tumors (n = 7 per group). *** *p* < 0.001; ** *p* < 0.01; * *p* < 0.05.

Next, we assessed the anti-tumor activity of the anti-TAG-72-IL-2 fusion protein against a syngeneic murine breast carcinoma. To study the effects of huCC49-IL-2 on syngeneic orthotopic tumors, we injected C57BL6 female mice into the mammary fat pad with E0771/TAG72 cells. When the tumors reached 100 mm^3^, the mice were treated with four daily doses (1 mg/kg) of huCC49-IL-2, administered via intraperitoneal (IP) injections. This dose was based on our previous study using anti-CEA M5A ICK [24]. We observed significant tumor growth inhibition starting on day 18 post tumor injection that lasted until day 25, when the experiment was terminated because the control tumors had reached their maximum allowed volume (Figure 3A).

The collected tissues were analyzed by flow cytometry to assess the immune phenotype of the major immune cell population infiltrates. At the terminal time-point analysis, when the treated tumor growth started to escape, we did not observe any changes in the T and NK cell infiltration in the huCC49-IL-2-treated tumors compared to the untreated controls (Figure 3B). Interestingly, in contrast to studies with anti-CEA M5A ICK, we did not observe a reduction in the T-reg fraction in the tumors, although some small changes were observed in the peripheral lymphoid tissues (Figure 3C). Nevertheless, we observed a significant increase in the IFNγ expression in both CD4- and CD8-positive T cells infiltrating tumors, which was attributed to the anti-tumor activity that slowed the tumor growth (Figure 3D).

**Figure 4:**
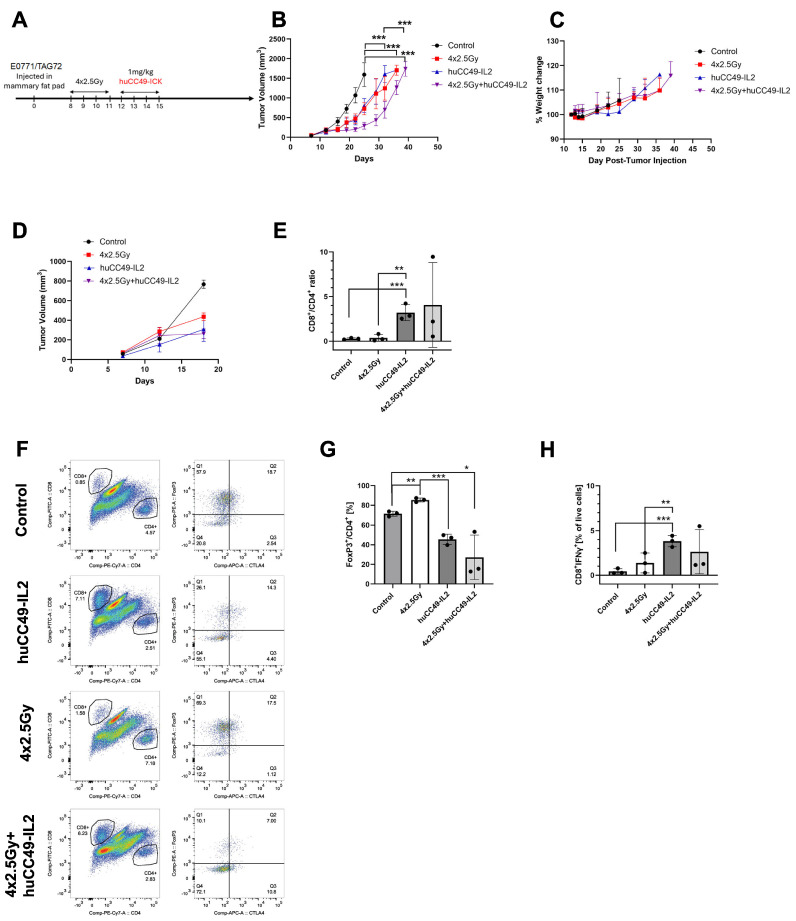
Combining radiation therapy with anti-TAG-72-IL-2 fusion protein in breast carcinoma model. (**A**) Study design scheme indicating tumor injection, tumor irradiation, and immunotherapy schedule. (**B**) Tumor growth kinetics in analyzed groups (n = 7 per group). (**C**) Whole-body weight changes in analyzed groups. (**D**) Tumor growth kinetics in mice used for immune phenotyping at day 18 post tumor injection (n = 3 per group). (**E**) Changes in CD8^+^ to CD4^+^ T-cell ratio in tumor tissues (n = 3 per group). (**F**) Representative flow cytometry dot plots of representative tumors showing CD8^+^ to CD4^+^ T-cell infiltration (left panels) and FoxP3 vs. CTLA-4 double staining of pre-gated CD4^+^ T cells (right panels). (**G**) Quantification of tumor-infiltrating FoxP3^+^ T-regs (n = 3 per group). (**H**) Changes in IFNγ-producing CD8^+^ T cells infiltrating tumor tissue shown as fraction of live cells (n = 3 per group). *** *p* < 0.001; ** *p* < 0.01; * *p* < 0.05.

Radiation therapy was combined with an anti-TAG-72-IL-2 fusion protein in a breast carcinoma model. In our previous study, we found that the combination of image-guided radiotherapy (IGRT) with anti-CEA M5A ICK was superior to monotherapy alone. Thus, we used a similar approach to that of anti-TAG-72 huCC49-IL-2, as shown in Figure 4A. In this model, fractionated IGRT had the same effect on tumor growth as huCC49-IL-2 monotherapy, but the combination therapy was significantly more effective and almost doubled the time required for tumor growth inhibition (Figure 4B). None of the therapies showed signs of whole-body toxicity, as shown by the absence of significant changes in the total body weight (Figure 4C). In this experiment, we selected three mice per group and collected tissues for immunophenotyping on day 18 post tumor injection, 3 days after the last dose of huCC49-IL-2. At that time point, each monotherapy slowed tumor growth kinetics, while the combination therapy group showed no tumor growth (Figure 4D). The flow cytometry analysis of the tumor tissues showed significant increases in CD8^+^ T cells in both groups treated with huCC49-IL-2, including significant increases in the CD8^+^ to CD4^+^ ratios (Figure 4E,F).

Moreover, both groups of tumors treated with huCC49-IL-2 showed significantly reduced T-reg populations within the tumor (Figure 4F,G). The IFNγ production was significantly elevated in both groups treated with huCC49-IL-2 (Figure 4H). In conclusion, huCC49-IL-2, alone or in combination with IGRT, induced strong anti-tumor immune responses, directly affecting tumor growth.

**Figure 5:**
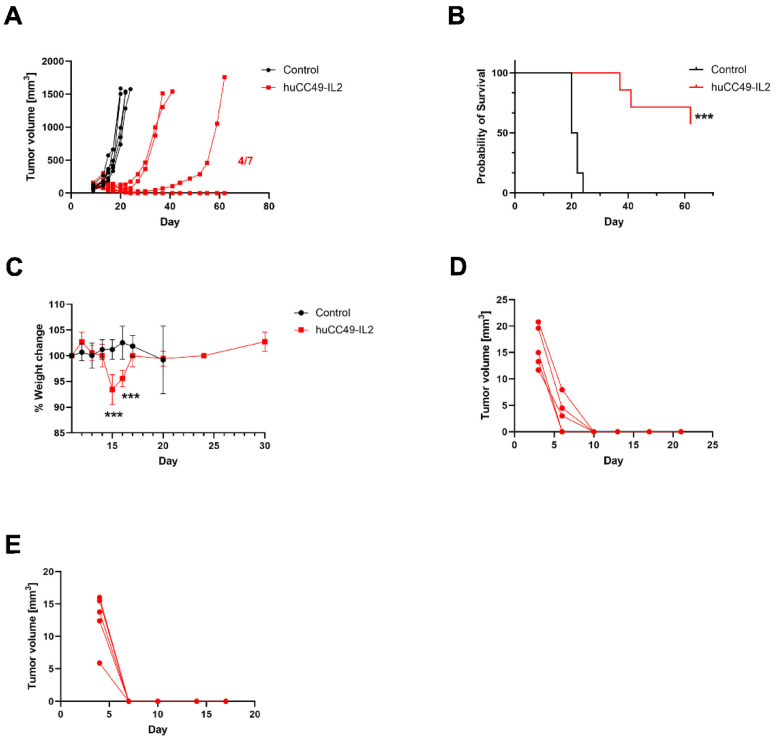
Anti-TAG-72-IL-2 fusion protein monotherapy of murine colon carcinoma. (**A**) Inhibition of MC38/TAG72 tumor growth. Tumor-bearing C57BL/6J male mice were treated with four daily doses of huCC49-IL2 (1 mg/kg) starting from day 11, when tumors reached 100 mm^3^ (n = 6–7 per group). (**B**) Survival curves for analyzed groups (n = 6–7 per group). (**C**) Whole-body weight changes in analyzed groups. (**D**) Tumor growth kinetics of MC38/TAG72 tumor rechallenge (n = 5 per group). (**E**) Tumor growth kinetics of MC38 parental tumor rechallenge (n = 5 per group). *** *p* < 0.001.

An anti-TAG-72-IL-2 fusion protein monotherapy of a murine colorectal carcinoma was conducted. To study the effects of the anti-TAG-72-IL-2 fusion protein in other TAG-72-positive tumor models, immunocompetent mice were injected subcutaneously with colorectal carcinoma syngeneic MC38 cells expressing cell surface TAG-72. On day 12, when the tumors reached 100 mm^3^ in volume, the mice were treated with four daily IP doses of huCC49-IL-2 (1 mg/kg), as in the previous experiments. All of the control tumors reached the maximum allowed volume by day 21 post injection, while four out of seven treated mice had eradicated primary tumors, and the remaining three had significantly slowed tumor growth (Figure 5A), a result that significantly extended their overall survival (Figure 5B). In this model, we observed temporal total body weight loss, which did not exceed 10% and recovered 2 days post therapy (Figure 5C). To assess the establishment of immune memory, five mice with the best primary tumor inhibition/eradication were rechallenged with a second tumor injection on the opposite flank. In all of the mice, the second tumor was rejected within 10 days post injection (Figure 5D). Furthermore, the same mouse group was rechallenged with MC38 parental cells at the same concentration 3 weeks after the MC38-TAG-72 rechallenge was rejected. In all of the mice, the second rechallenge tumor was rejected within 7 days (Figure 5E). This result suggested that delivering the anti-TAG-72-IL-2 fusion protein to the tumor resulted in the generation of immune memory and antigen spreading against antigens common to both parental and TAG-72-expressing MC38 cells.

**Figure 6:**
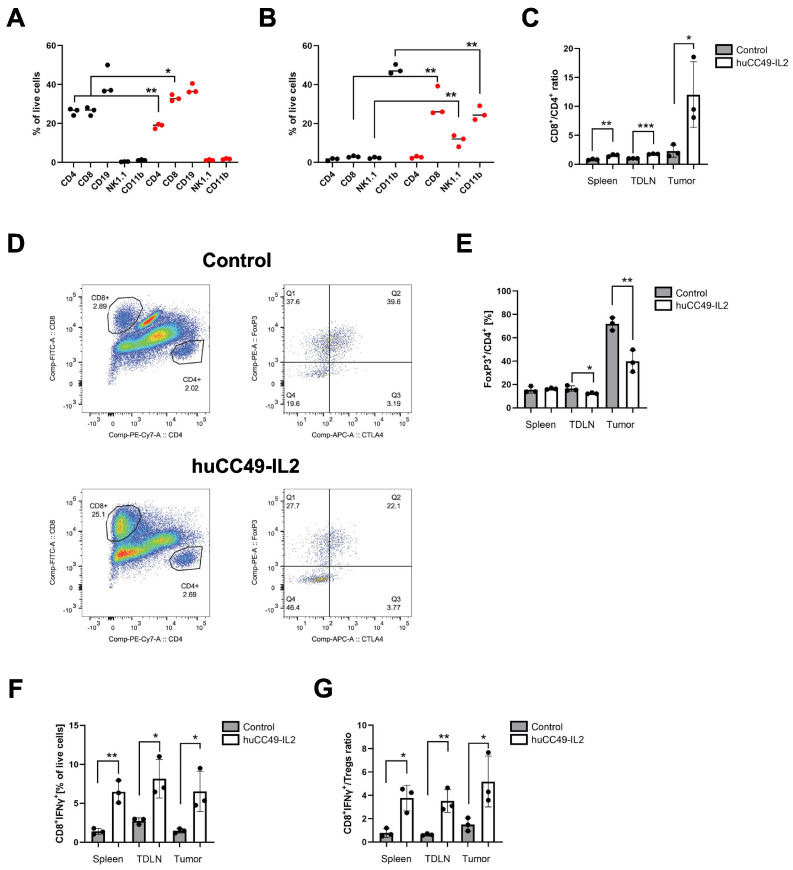
Potent infiltration and activation of CD8^+^ T cells post huCC49-IL-2-fusion protein treatment. (**A**) Flow cytometry analysis of tumor-draining lymph nodes (black control, red huCC49-IL-2, n = 3 per group). (**B**) Flow cytometry analysis of tumor-infiltrating immune cell subsets (black control, red huCC49-IL-2, n = 3 per group). (**C**) Changes in CD8^+^ to CD4^+^ T-cell ratio in indicated tissues (n = 3 per group). (**D**) Representative flow cytometry dot plots of representative tumors showing CD8^+^ and CD4^+^ T-cell infiltration (left panels) and FoxP3 vs. CTLA-4 double staining of pre-gated CD4^+^ T cells (right panels). (**E**) Quantification of tumor-infiltrating FoxP3^+^ T-regs in indicated tissues (n = 3 per group). (**F**) Changes in IFNγ-producing CD8^+^ T cells in indicated tissues shown as fraction of live cells (n = 3 per group). (**G**) Changes in IFNγ^+^CD8^+^/Treg ratios in indicated tissues (n = 3 per group). *** *p* < 0.001; ** *p* < 0.01; * *p* < 0.05.

We next evaluated the changes in the immune cell tumor infiltration at early time points. We collected tissues at day 18, 3 days after the last dose of huCC49-IL-2. At that time, the huCC49-IL-2-treated tumor volumes started to decrease (Supplementary Figure S2A). The flow cytometry analysis of the tumor-draining lymph nodes showed a significant expansion of CD8^+^ T cells and a decrease in CD4^+^ T cells (Figure 6A), whereas in the spleen, we observed similar changes in the CD8^+^ population (Supplementary Figure S2B). A similar analysis of the tumor tissues showed a massive and significant increase in the CD8^+^ T-cell infiltration, followed by an increased number of NK cells (Figure 6B). The results indicated significantly altered proportions of primary T-cell subtypes, favoring cytotoxic CD8^+^ populations in lymphoid tissues, particularly in tumors (Figure 6C).

Similar to what we observed in the mammary tumor model (Figure 4F,G), we found a large reduction in the regulatory CD4^+^ T cells (Figure 6D,E). To determine the activation status of the cytotoxic T cells, we analyzed the IFNγ expression by the intracellular flow cytometry of CD8^+^ T cells. We found a significant increase in the IFNγ-expressing CD8^+^ T cells, both in the periphery and in the tumors (Figure 6F). Altogether, the huCC49-IL-2 treatment resulted in the generation of a potent anti-tumor immune environment, as evidenced by the changes in IFNγ^+^CD8^+^ T to CD4^+^ T-reg ratios (Figure 6G).

## 4. Discussion

Targeting tumor antigens with immunocytokines designed to boost anti-tumor immunity has been of increasing interest since the early 2000s [27]. Interleukin 2-based fusion proteins have been intensively tested in both the pre-clinical and early clinical phases [28]. These antibody fusion proteins have been engineered to target several different tumor antigens [29]. One of the most abundant and highly specific tumor antigens expressed in several epithelial cancers is TAG-72. TAG-72 expression is associated with a poor prognosis in colorectal and hepatocellular carcinomas [30,31].

The feasibility and biological activity of the IL-2 fusion molecule targeting TAG-72 was previously tested by Shu et al. using an engineered single-chain immunoglobulin-interleukin-2 fusion protein (SCIg-IL-2) derived from the chimeric CC49 antibody [32]. However, the study was limited to an in vitro characterization of the molecule and its bioactivity and cytolytic antibody-dependent cell-mediated cytotoxicity (ADCC) function when compared to the single-chain antibody SCIgACnl. In this study, we report the generation of the first fully humanized anti-TAG-72 IL-2 fusion protein. We confirmed its specificity and anti-tumor activity as monotherapy or in combination with stereotactic radiation in syngeneic models with intact immune systems analogous to clinical applications. This study required the use of murine carcinoma cell lines expressing a human target antigen. Therefore, cells were engineered to express the target antigen TAG-72 by transduction with the epHIV7 lentivirus carrying the murine *st6galnac-I* gene (mSTn) under the control of EF1α. mSTn is a unique sialyltransferase that generates the surface expression of aberrant glycosylation sialyl-Tn (TAG72) [33]. In both tested tumor models (breast and colon carcinomas), spontaneous tumor rejection was not observed in C57BL/6J mice (same background as tumor cells), indicating that TAG-72 is not necessarily immunogenic. This genetic construct was used by the Priceman group in the City of Hope to evaluate CAR-T therapy in an ovarian cancer model that showed the value of a clinically relevant model that progressed into a phase I clinical trial [4].

TAG-72 is expressed in a high proportion of breast cancers and is significantly expressed in a subgroup of patients with a larger tumor size, lymph node metastasis, and high histopathological grade [34]. Therefore, we tested huCC49-IL-2 in a highly aggressive E0771 mammary carcinoma model. Although monotherapy showed significant tumor growth inhibition, the tumors started to regrow earlier than those in our similar study using anti-CEA M5A ICK therapy against CEA-expressing E0771 tumors [24].

In this study, the best results of the huCC49-IL-2 monotherapy were observed in the MC38/TAG72 colorectal model. Similar better responses to the huCC49-IL-2 treatment were found in MC38/CEA vs. E0771/CEA tumors, which were attributed to very different myeloid cell compositions in each of these tumor models [24]. In the current study, using tumor cells overexpressing TAG-72, we found similar myeloid cell subpopulations with predominantly macrophages in MC38/TAG72 tumors vs. high Ly6 monocytic cells in the E0771/TAG-72 tumors. Moreover, in the MC38/TAG72 model, four out of seven mice had eradicated primary tumors as well as second tumor challenges, indicating the establishment of immune memory. The early time-point post-treatment analysis indicated massive CD8^+^ T-cell infiltration and activation. Furthermore, the tumor microenvironment was completely reshaped toward anti-tumor inflammatory cells, as illustrated by changes in the CD8 to CD4 ratio and IFNγ-producing CD8^+^ T cells to T-regs ratio in the huCC49-IL-2-treated tumors and lymphoid organs (Figure 6G). We believe that these changes are critical for achieving potent and lasting anti-tumor immune responses, as shown previously by us and others [24,35].

Immunotherapies differ from conventional chemotherapy in several ways, as they do not directly target or kill tumor cells. Instead, these therapies stimulate the immune system to produce an immune response against tumors. A consequence of immunotherapy could be the release of secondary tumor antigens, called antigen spreading, which can become a source of new targets for immune cells [36,37]. It was previously reported that mice vaccinated with a CEA-based vaccine showed significant decreases in the growth of both CEA^+^ and CEA^–^ tumors [38]. In our therapy study targeting TAG-72 in the MC38/TAG72 colorectal model, we observed not only an immune response against the tumor rechallenge with the same TAG-72-expressing cells but also against the parental cell line (Figure 5D,E). This finding provides strong evidence that delivering IL-2 to the primary tumor causes the immune system-driven generation of secondary antigens shared by parental and TAG-72-overexpressing MC38 cells.

## 5. Conclusions

In summary, we have shown that a humanized anti-TAG-72-IL-2 fusion protein achieved optimal tumor targeting and target specificity and induced immune responses in animal models. As a result, not only did tumor growth diminish, but we also observed prolonged tumor immunity. Future clinical testing in different TAG-72-positive tumors is warranted, particularly when combined with other modalities such as image-guided radiotherapy.

## Data Availability

The data are available on reasonable request by contacting the corresponding author (M.K.).

## Supplementary Materials

The following supporting information can be downloaded at https://www.mdpi.com/article/10.3390/cancers17091453/s1, File S1: The original gels; Figure S1: Genetic constructs of huCC49-IL-2; Figure S2: Early time point analysis of anti-TAG-72 ICK treated murine colorectal carcinoma.

## Abbreviations

ADC	Antibody–drug conjugate
ADCC	antibody-dependent cell-mediated cytotoxicity
CAR-T	chimeric antigen receptor T cells
CEA	carcinoembryonic antigen
CHT column	ceramic hydroxyapatite
CTLA-4	cytotoxic T-lymphocyte associated protein 4
DNA	deoxyribonucleic acid
E0771	murine breast cancer cell line
FoxP3	transcription factor forkhead box P3
HC	heavy chain
huCC49	humanized anti-tumor associated glycoprotein
ICK	Immunocytokine
IFNg^+^	interferon gamma
IGRT	image-guided radiation therapy
IL-2	interleukin
IL-2R	interleukin-2 receptor
IP	intraperitoneal
M5A	humanized anti-CEA antibody
MBq	megabecquerel
MC38	murine colon adenocarcinoma cell line
mSTn	murine st6galnac-1 gene
Nacl	sodium chloride
NK	natural killer cell
OV90	human ovarian carcinoma
PBS	phosphate-buffered saline
PET	positron emission tomography
PD-1	programmed cell death protein 1
SCIg-IL-2	single-chain immunoglobulin-interleukin-2 fusion protein
SDS-PAGE	sodium dodecyl-sulphate polyacrylamide gel electrophoresis
TAG-72	tumor-associated glycoprotein 72
TAT	targeted alpha therapy
