# Supplementary material for: Development and Potent Anti-Tumor Activity of a Fully Humanized Anti-TAG-72-IL-2 Fusion Protein for Therapy of Solid Tumors
Source: Cancers (Basel). 2025 Apr 26;17(9):1453. doi: 10.3390/cancers17091453 (PMC12071099; doi:10.3390/cancers17091453)
Supplement: Supplementary file 1 [file cancers-17-01453-s001.zip › Supplementary Figure S1.pdf]

A

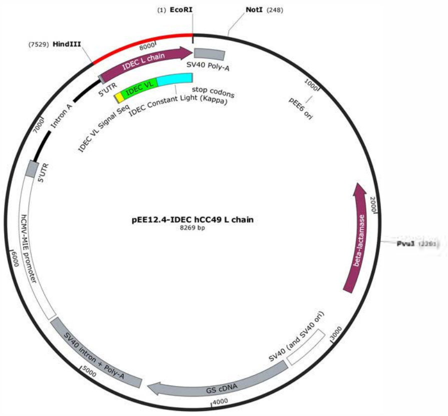

B

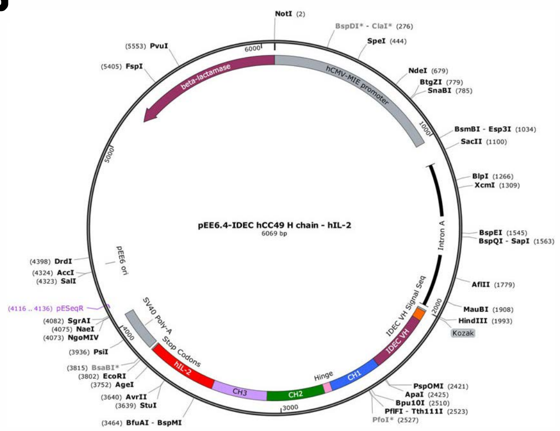

C

Heavy Chain

*MGWSLILLFLVAVATRVLS*QVQLVQSGAEVVKPGASVKISCKASGYTFTDHAIHWVKQNPGQRLEWIGYFSPGNDDFK
YNERFKGKATLTADTSASTAYVELSSLRSEDTAVYFCTRSLNMAYWGQGTLVTVSSASTKGPSVFPLAPSSKSTSGGTAAL
GCLVKDYFPEPVTVSWNSGALTSGVHTFPAVLQSSGLYSLSSVVTVPSSSLGTQTYICNVNHKPSNTKVDKKVEPKSCDK
THTCPPCPAPELLGGPSVFLFPPKPKDTLMISRTPEVTCVVVDVSHEDPEVKFNWYVDGVEVHNAKTKPREEQYNSTYR
VVSVLTVLHQDWLNGKEYKCKVSNKALPAPIEKTISKAKGQPREPQVYTLPPSRDELTKNQVSLTCLVKGFYPSDIAVEWE
SNGQPENNYKTTPPVLDSDGSFFLYSKLTVDKSRWQQGNVFSCSVMHEALHNHYTQKSATATPG**APTSSSTKKTQLQL
EHLLLDLQMILNGINNYKNPKLTRMLTFKFYMPKKATELKHLQCLEEELKPLEEVLNLAQSKNFHLRPRDLISNINVIVL
ELKGSETTFMCEYADETATIVEFLNRWITFCQSIISTLT**

Leader sequence, Heavy chain, CDRs underlined, in bold **IL-2**

Light chain

*MDSQAQVLMLLLLWVSGTCG*DIVMSQSPDSLAVSLGERVTLNCKSSQSLLYSGNQKNYLAWYQQKPGQSPKLLIY
WASARESGVPDRFSGSGSGTDFTLTISSVQAEDVAVYYCQQYYSYPLTFGAGTKLELKRTVAAPSVFIFPPSDEQLKSGTAS
VVCLLNNFYPREAKVQWKVDNALQSGNSQESVTEQDSKDSTYSLSSTLTLSKADYEKHKVYACEVTHQGLSSPVTKSFNR
GEC

Leader sequence, Light chain, CDRs underlined

Supplementary Figure S1. Genetic constructs of huCC49-IL-2. Genetic maps of pEE12.4 plasmid vectors of a light chain **(A)** and a heavy chain-IL2 fusion **(B)**. **C.** Protein amino acids sequences. Leader sequence (italic), complementarity determining regions (CDRs - underlined), IL-2 (bold).
