# Supplementary material for: Development and Potent Anti-Tumor Activity of a Fully Humanized Anti-TAG-72-IL-2 Fusion Protein for Therapy of Solid Tumors
Source: Cancers (Basel). 2025 Apr 26;17(9):1453. doi: 10.3390/cancers17091453 (PMC12071099; doi:10.3390/cancers17091453)
Supplement: Supplementary file 1 [file cancers-17-01453-s001.zip › Supplementary Figure S2.pdf]

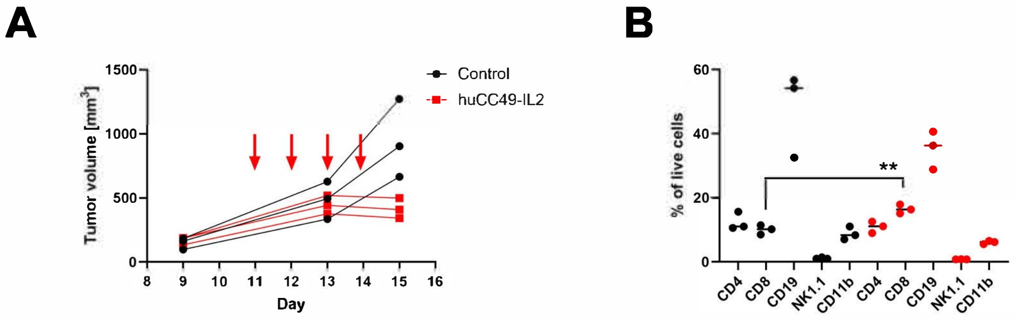

Supplementary Figure S2. Early time point analysis of anti-TAG-72 ICK treated murine colorectal carcinoma.
A. Tumor growth kinetics in mice used for immune phenotyping at day 18 post tumor injection (n=3 per group).
B. Flow cytometry analysis of immune subsets the spleens (black Control, red ICK, n=3 per group) ** $p<0.01$.
