# Supplementary figures and images for: Development and Potent Anti-Tumor Activity of a Fully Humanized Anti-TAG-72-IL-2 Fusion Protein for Therapy of Solid Tumors
Source: Cancers (Basel). 2025 Apr 26;17(9):1453. doi: 10.3390/cancers17091453 (PMC12071099; doi:10.3390/cancers17091453)

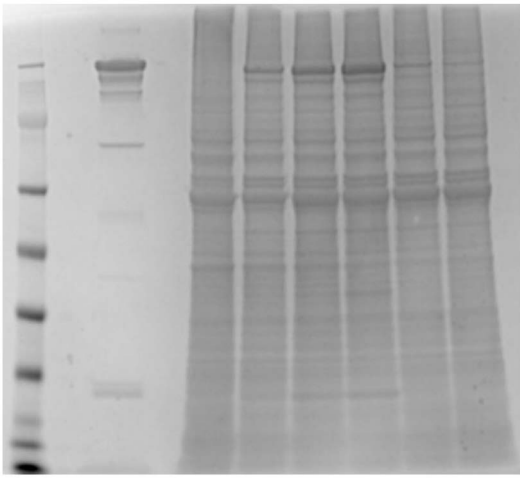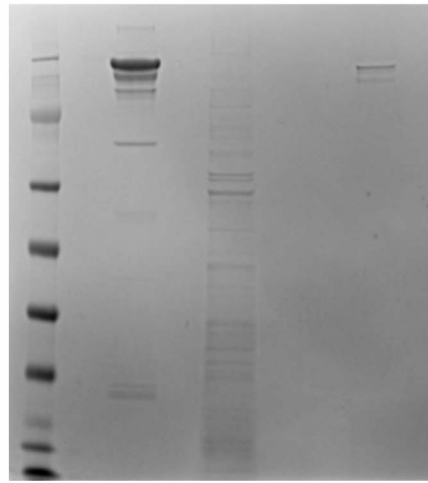

**Original figures of gels from Figure 1**

Supplement: Supplementary file 1 [file cancers-17-01453-s001.zip › Supplementary File S1.pdf]
